# Diffusion of Nitrogen and Phosphorus Across the Sediment-Water Interface and In Seawater at Aquaculture Areas of Daya Bay, China

**DOI:** 10.3390/ijerph110201557

**Published:** 2014-01-28

**Authors:** Xiangju Cheng, Yingxue Zeng, Zhenren Guo, Liangsheng Zhu

**Affiliations:** 1 School of Civil Engineering and Transportation, South China University of Technology, Guangzhou 510640, China; E-Mails: z.yingxue@mail.scut.edu.cn (Y.Z.); lshzhu@scut.edu.cn (L.Z.); 2 Faculty of Engineering, Institut Teknologi Brunei, Gandong BE 1410, Brunei; E-Mail: guozhenren@scies.org; 3 South China Institute of Environmental Sciences, MEP, Guangzhou 510655, China

**Keywords:** sediments, nitrogen and phosphorus, aquaculture, diffusion, Daya Bay

## Abstract

With the yearly increasing marine culture activities in floating cages in Daya Bay, China, the effects of pollution may overlap and lead to more severe water environmental problems. In order to track the impacts of the marine culture in floating cages on water environment, sediments and overlying water were sampled by cylindrical samplers at three representative aquaculture areas of Daya Bay. The water content, porosity, density of sediments as well as the vertical distributions of ammonia nitrogen and active phosphate in pore water along sediments depth were measured. The release rate and annual released quantity of the nutrients across sediment-water interface were calculated using Fick’s Law. A horizontal two-dimensional mathematical model was developed to compute the spatial and temporal distributions of the nutrients in seawater after being released across the sediment-water interface. The results showed that the sediments, with a high content and a large annual released quantity of nitrogen and phosphorus, constitute a potential inner source of seawater pollution. Influenced by tide and water depth, the scope of diffusion and migration of the nutrients appears as a long belt which is about 1 km long and 50 m wide. Seawater in this area is vulnerable to eutrophication.

## 1. Introduction

As both a source and sink of nitrogen and phosphorus in different water bodies, sediments influence the process of eutrophication as well as the restoration and governance of water quality. When nutrients from outer sources are discharged into water bodies, a great deal of nitrogen and phosphorus accumulates in sediments and their concentrations may be up to 50 to 500 times that in the overlying water [1]. The water environment is under potential threat, due to such a great amount of nitrogen and phosphorus being released across the sediment-water interface and transported into water bodies.

As a semi-enclosed drowned-valley bay with both tropical and subtropical characteristics, Daya Bay was listed as a key economic development zone of Guangdong (China) in 1984 [2]. With the increasing demand for seafood, large-scale marine aquaculture, especially the marine culture in floating cages, has been developed. The residual baits, metabolites and excreta from aquaculture deposit in and enrich the sediments. They are released again under certain conditions, polluting the water environment and becoming the most important inner source of nitrogen and phosphorus [3,4]. At present, there are lots of laboratory experiments and prototype observations about the release and diffusion of nutrients in lakes [5], estuaries [6], and wetlands [7]. However, they are mainly concerned with the release of nutrients which happens at the sediment-water interface. In fact, the process and extent of transport and diffusion of the nutrients, as well as the scope of pollution that leads to the eutrophication in water bodies are still problems that concern water environment management departments a lot. Influenced by the topography, tide and wind, water exchange in Daya Bay is extremely non-fluent [8], a phenomenon that makes the nutrients easily accumulate in water bodies. Considering the above, this study aims to understand the whole diffusion process of nutrients from the sediments to sea water and will supply a theoretical basis for managing the ocean environment. We attempted to sample sediments and overlying water *in situ* at aquaculture areas of Daya Bay and determine the vertical distributions in sediment and transport contents of nitrogen and phosphorus across the water-sediment interface. Based on the theory describing transport and diffusion of environmental water contaminants, the degree of impacts from aquaculture sediments on the water environment in Daya Bay is calculated and analyzed and the spatial distribution characteristics of nutrient concentrations in seawater are figured out.

## 2. Survey and Methods

**Figure 1:**
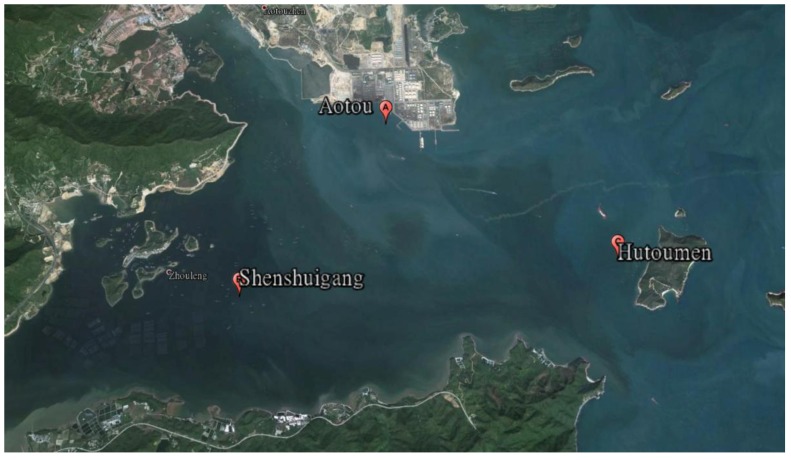
Locations of the three sample sites.

Aotou (22°42′49.3′′N, 114°32′31.76′′E), Shenshuigang (22°41′49.5′′N, 114°32′23.8′′E), and Hutoumen (22°40′12.5′′N, 114°36′9.9′′E) in Daya Bay were chosen as sampling sites (see their location in Figure 1). There are about 100 sets of floating aquaculture cages near the three sites, mainly breeding red grouper (*Epinephelus akaara*), grey mullet (*Mugil cephalus*), and mud crab (*Scylla serrata*) and so on. The baits used are mostly fresh small trash fish and artificial baits. Varying by season and water temperature, the average daily feeding bait dose is about 3% to 10% of the fishes’ weight. The survey date was collected early in October 2011.

**Figure 2:**
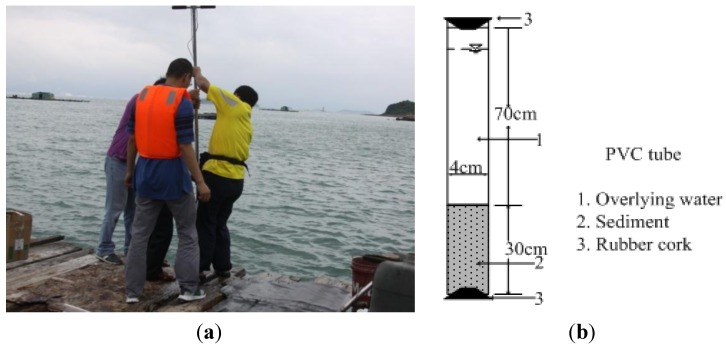
(**a**) One of the photos taken during field sampling; (**b**) Sketch of the PVC tube.

Overlying water and sediments were sampled by a cylindrical sampler connected a PVC tube at the three representative sites. The cylindrical sampler connected a PVC tube was inserted into sediments through the action of gravity. The top of sampling tube was sealed by gravity and pressure after the sampler stopping moving downward. In order to prevent the sediments in the sampling tube from falling off, a rubber cork was used to block the bottom of the tube when the sampler was to be pulled out of the water surface. This sampling process ensures the original structure of sediment in seawater undisturbed. Depending on the needs of the parallel laboratory experiments, about 30 PVC tubes were used to contain the sediments and overlying water, *i.e.*, 10 PVC tubes were used to collect sediments and overlying water at each site. Each PVC tube has an inside diameter of 4 cm and a height of 1 m. After sampling, the samples were transported to the laboratory immediately for processing and measuring. A photo taken during field sampling and a sketch of the PVC tubes are shown in Figure 2. As seen from Figure 2a, our sample collectors stood on a floating cage and used the gravity cylindrical sampler for vertically collecting sediment samples and overlying water. As seen from Figure 2b, the sampled sediments and overlying water were contained in the PVC tube, and the height of PVC tube is about 1 m, the contained sediment is 30 cm in height and the overlying water is 60 cm in height and the free space is 10 cm in height. 

Table 1 shows the measured parameters of the water body in Daya Bay during this investigation. The variable *T* means water temperature, DO is the concentration of dissolved oxygen in seawater, *S* is the salinity, and *H* is water depth. After being transported to the laboratory, the sediment columns within 5 cm from the surface were cut into 1 cm height each, the rest were cut into 2 cm pieces each. The contents of nitrogen and phosphorus in each small sediment slice were approximately considered to be uniformly distributed. The small sediment slices which could not be measured in time were put into glass bottles and stored in a refrigerator at a temperature of 4 °C. At each sampling site, several sediment columns were selected to be cut, placed on polyethylene bags, dried naturally, grinded, sieved by a 100-mesh sieve, and put into jars for later use.

Each small sediment slice was centrifuged at 3,000 rpm for 30 min. After separating with solids, the pore water was placed in a glass bottle and stored in a refrigerator. The moisture content in the sediment was determined by a gravimetric method. The porosity of sediments was the volume ratio of pore water and the sediment. The density of sediment is the bulk density, expressed by the weight per volume of sediment. The content of total phosphorus in sediments was determined by the microwave digestion-phosphorus vanadium molybdenum yellow spectrophotometry method. The determination of NH_4_^+^-N in sediments was performed by an extraction with potassium chloride solution-spectrophotometric method. The Kjeldahl nitrogen content in sediment was measured by the potassium persulfate oxidation method. The concentration of NH_4_^+^-N in pore water and overlying water was determined by the Nessler reagent spectrophotometric method, and the dissolved reactive phosphorus (DRP) by the phosphomolybdenum blue method. The principles of measurement for nutrients can be found in “*The Specification for Marine Monitoring*” (GB17378.4-1998) protocols. Three replicate measurements were done for each parameter and from each site. Due to the short measurement time, the temperature, DO and salinity at the bottom and surface of each water column remained constant.

**Table 1:** Parameters field-observed in Daya Bay.

Site	*pH*	*T*/°C	DO/(mg L^−1^)	*S*/‰	*H*/m
Aotou	8.49	27.04	5.64	31.63	≈7.5
Shenshuigang	8.54	26.51	7.32	31.76	≈8.2
Hutoumen	8.39	26.76	6.22	32.98	≈9

## 3. Results and Discussion

### 3.1. Physical Parameters of Sediments

**Figure 3:**
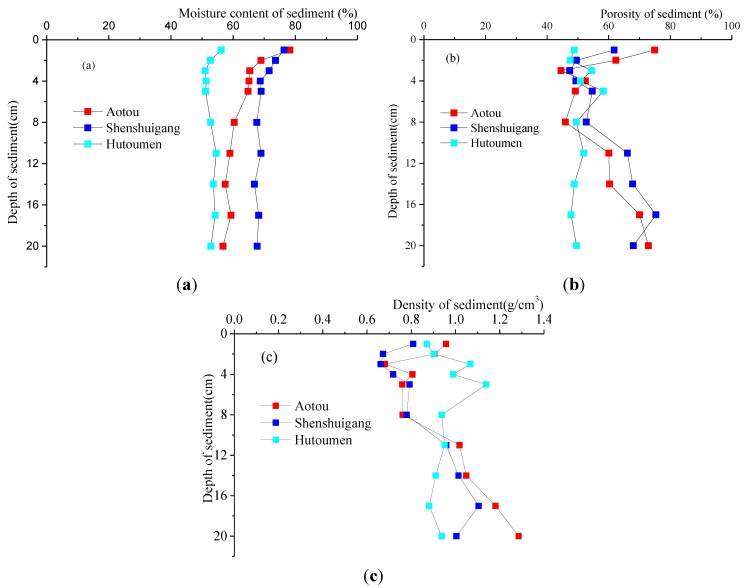
Vertical distributions of physical parameters in sediments.

The vertical distributions of physical parameters in the sediments from aquaculture areas of Daya Bay are shown in Figure 3. A seen in Figure 3a, we can find that the moisture contents of sediments in Aotou, Shenshuigang and Hutoumenare are 56.7%–78.3%, 67.7%–76.4%, and 51.0%–56.1% respectively. They all decrease with increasing depth and finally tend to a constant value. As seen from Figure 3b, the porosities of sediments in Aotou, Shenshuigang and Hutoumen are 44.5%–74.9%, 57.3%–75.3%, and 47.4%–51.9% respectively. Because of the sufficient contact between the sediments and overlying water, as well as the active biogeochemical dynamics across the sediment-water interfaces, the porosities of sediments in these three sites are all large on the surfaces. As seen from Figure 3c, the densities of sediments in Aotou, Shenshuigang and Hutoumen are 0.68–1.28 g/cm^3^, 0.66–1.10 g/cm^3^, 0.87–1.14 g/cm^3^ respectively. They all increase with increasing depth. 

### 3.2. Nitrogen and Phosphorus Contents in Sediments

**Figure 4:**
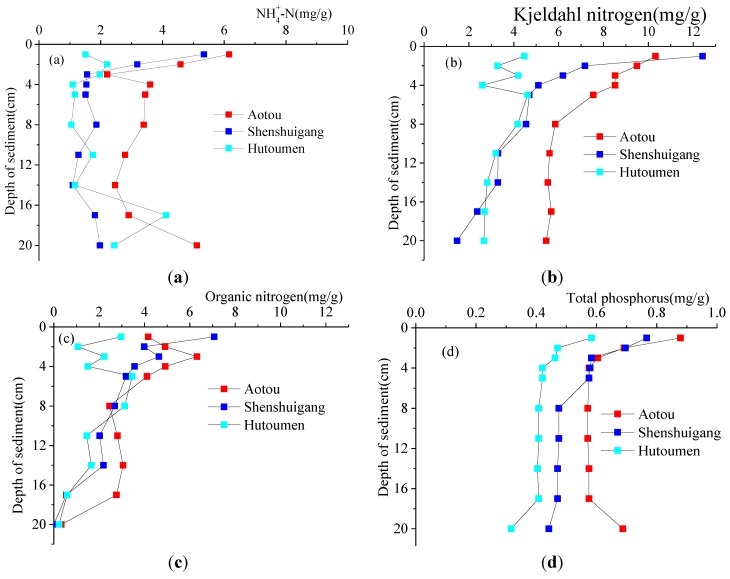
The vertical distributions of nitrogen and phosphorus in sediments.

The vertical distributions of nitrogen and phosphorus in the sediments are shown in Figure 4. From Figure 4, we can find that nitrogen and phosphorus contents in the sediments decrease with increasing depth and finally stabilize. The active area of nitrogen and phosphorus is on the surface of the sediments within 4 cm vertical depth. Given this, the area within 4 cm vertical depth along the sediments was considered mainly when we analyzed the release and transport of nutrients across the sediment-water interface. As shown in Figure 4, the variation of Kjeldahl nitrogen content in the sediments are almost the same in the three sites, and Kjeldahl nitrogen content decreased with increasing depth. The degrees of change of Kjeldahl nitrogen in Hutoumen and Aotou become smaller and smaller with the increasing depth of sediments. However, that in Shenshuigang remains rather large within the area we measured. We forecast that it will slow down with the further increasing depth. Generally speaking, Kjeldahl nitrogen is equal to the sum of organic nitrogen and ammonia nitrogen. According to the contents of ammonia nitrogen and Kjeldahl nitrogen determined in the three sites, the vertical distribution of organic nitrogen in the sediments can be calculated easily as shown in Figure 4c. The larger the depth, the smaller the content of organic nitrogen, which eventually tends to 0, indicating that organic nitrogen distributes mainly on the surface of the sediments. Ignoring the abnormal points in Figure 4d, the content of total phosphorus in the sediments decreases with increasing depth and finally stabilizes. 

### 3.3. Distributions of Nitrogen and Phosphorus in Pore Water

**Figure 5:**
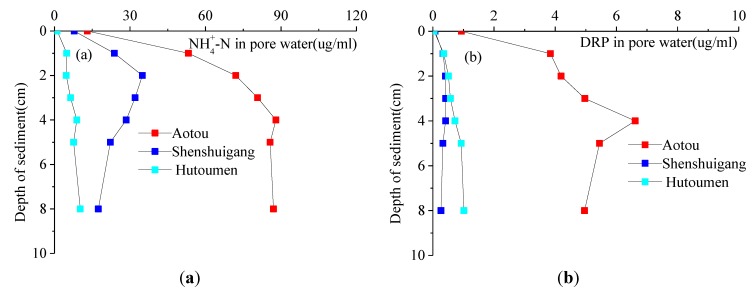
Vertical distributions of N, P in pore water of sediments.

Many forms of nitrogen exist in pore water, such as ammonia nitrogen, nitrate nitrogen, nitrite nitrogen and some small-molecule organic compounds. As for phosphorus, it exists mainly in dissolved reactive phosphate (DRP) form. Although the distributions of nitrate nitrogen, nitrite nitrogen and ammonia nitrogen (NH_4_^+^-N) have been determined in detail, in order to reduce the length of this article, only the vertical distributions of NH_4_^+^-N and DRP in pore water are discussed, as shown in Figure 5. At these three sites, the concentrations of NH_4_^+^-N in pore water all become higher with increasing depth and finally stabilize. The vertical gradient of NH_4_^+^-N concentration contributes to the diffusion of NH_4_^+^-N from the pore water of sediments to the water body. The generation and whereabouts of NH_4_^+^-N in sediments are influenced and restricted by a variety of factors, such as the pollution level, the extent of biological effects, redox state, hydrodynamic impact and so on [9]. In polluted waters like Aotou, sediments are so rich in organic matter that there are a great number of microbes on their surface. The more significant biological decomposition in Aotou compared with Hutoumen results from a hypoxia, which easily forms a reducing environment. Due to the obvious biological denitrification and ammonification [10], more NH_3_ is admitted into the interstitial water on the surface of sediments. 

Compared the contents of NH_4_^+^-N measured in this study and Huang *et al.* [11] in the surface of sediments at the sample site of Hutoumen, the measured data are near close to each other. However, as seen from the Figure 1, because the sample site of Aotou is close to a little town, where the population is dense, much domestic sewage and industry waste water are discharged into the ocean area, which not only results in malodorous, black sea water, but also the color of the collected sediment looks black and is smelly. The sample sites of Shenshuigang and Hutoumen are from little towns about 5 km and 7 km away, respectively. In the sea area of Shenshuigang there are more floating cages than in the Hutoumen area, so the nitrogen and phosphorus contents at Aotou are significantly higher than those of the other two stations and the nitrogen and phosphorus contents at the Shenshuigang are higher than those of Hutoumen.

The DRPs in pore water of the sediments in Aotou, Shenshuigang and Hutoumen are 0–7 μg/mL, 0–3 μg/mL, and 0–1 μg/mL, respectively. The concentration of DRP in pore water increases to a certain value and then decreases with increasing depth and finally tends to a relatively stable value. The concentration of DRP among the sediments in Aotou is the highest, followed by that in Shenshuigang, and the minimum is in Hutoumen, indicating that DRP from sediments in Aotou is more easily released into the overlying water. The concentration gradient is the main determinant of the nutrient release. Generally speaking, the higher the concentration gradient in pore water, the higher the release rate. At a depth of about 6 cm, the concentration variations of DRP in the three sites gradually level off or even decrease, since the sediments here are relatively dense and lack an active organic detritus layer [12].

**Figure 6:**
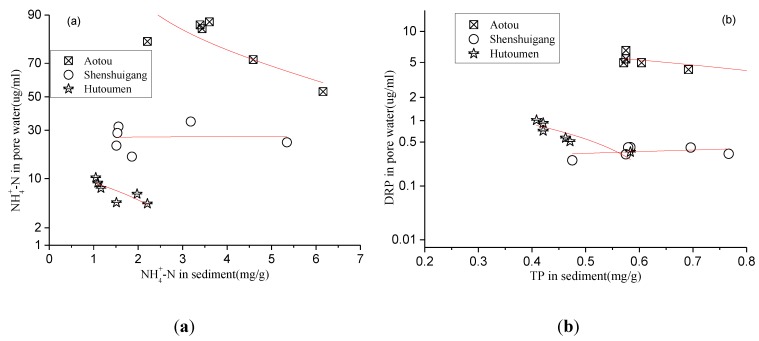
Relationship of N, P contents between in pore water and in sediments.

The comparison between the contents of NH_4_^+^-N and DRP in pore water and the NH_4_^+^-N and TP in sediments at three sample sites is shown in Figure 6(a,b). As seen from Figure 6, a certain inverse relationship of the nutrient contents between in pore water and in sediments is observed at the sample sites of Aotou and Hutoumen, which means that the nutrient contents stored in sediments are the nutrient content determinants in pore water. However, there is low correlation of nutrient contents between in pore water and in sediments at the sample site of Shenshuigang, which means there may be some unknown factors controlling release at this site. 

### 3.4. Diffusion Flux of Nutrients across Sediments-Water Interface

Due to the ever-increasing loads of nitrogen and phosphorus from aquaculture, lots of dissolved or granular nitrogen and phosphorus accumulate on the surface of the sediments by flocculation, adsorption and sedimentation, resulting in growing contents of nitrogen and phosphorus in the overlying sediments. This indicates that in Daya Bay, especially near the aquaculture-cages, a large amount of nitrogen and phosphorus from the sediments is released into the overlying water. It can be seen from the above analysis that there are concentration gradients of nitrogen and phosphorus in pore water along the sediment depth. The diffusion fluxes of NH_4_^+^-N and DRP across sediment-water interface can be computed by Fick’s first law [13] shown as Equation (1):

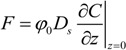
(1)
where *F* is the diffusion flux across sediment-water interface, *C* is the concentration of nutrients,*φ*_0_is the porosity of sediments on the surface, 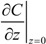 is the concentration gradient of nutrients across sediment-water interface, and *D*_s_ is the actual molecular diffusion coefficient with consideration of bending effect in sediments. It is very difficult to measure the curvature of the sediments. Ullman *et al*. [14] propose an empirical equation relating the actual diffusion coefficient *D*_s_ and porosity *φ*_0_:

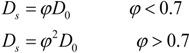
(2)
where *D*_0_ is the ideal diffusion coefficient of nutrients in the infinitely dilute solution. For DRP, *D*_0_ = 7.0 × 10^−6^ cm^2^∙s^−1^. For NH_4_^+^-N, *D*_0_ = 17.6 × 10^−6^ cm^2^∙s^−1^.

Equation fitting relating nutrients content in pore water to sediments depth was conducted and is shown in Table 2 and Table 3. Then we took the derivative of depth *z* (*z* is the positive vertical depth and *z = 0* represents the sediment-water interface) and obtained the value of 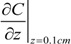 across the sediment-water interface (note the concentration gradient of nutrients was seat at *z = 0.1* cm because it is impossible to make *z = 0* to be on the ideal sediment-water interface).

Judging from Table 2 and Table 3, the fitting results for the three sites are all desirable, with multiple correlation coefficients R^2^ > 0.98, indicating that it is reasonable to use this kind of fitting curves to calculate the concentration gradient of nutrients. According to the porosity determined above, the area at a depth of 0–4 cm is an active one for the sediments, so the average porosity of the sediments in this area was discussed. Based on Figure 3, the average porosities in the active areas for three sites can be computed as follows: 58.5% for Aotou, 52% for Shenshuigang, and 50.3% for Hutoumen, which are less than 70%. Using Equation (2), the actual diffusion coefficients of nutrients in three sites were acquired. It can be found from the field investigation that the areas which are influenced by aquaculture in these sites are about 100 m^2^. The diffusion fluxes and release quantities of nutrients in three sites were calculated as shown in Table 4.

**Table 2:** Fitting equations related the NH_4_^+^-N concentration to vertical depth on sediment-water interface in Daya Bay.

Site	NH_4_^+^-N		
	Fitting Equation	*R* ^2^	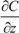 /(μg∙mL^−1^∙cm^−1^)
Aotou	*C* = 58.015*z* − 12.213*z*^2^ + 0.791*z*^3^	0.993	55.596
Shenshuigang	*C* = 30.849*z* − 8023*z*^2^ + 0.581*z*^3^	0.984	29.220
Hutoumen	*C* = 4.245*z* − 0.792*z*^2^ + 0.053*z*^3^	0.981	4.088

**Table 3:** Fitting equations related the DRP concentration to vertical depth on sediment-water interface in Daya Bay.

Site	DRP		
	Fitting Equation	*R* ^2^	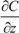 /(μg∙mL^−1^∙cm^−1^)
Aotou	*C* = 3.622*z* − 0.761*z*^2^ + 0.048*z*^3^	0.985	3.471
Shenshuigang	*C* = 0.379*z* − 0.096*z*^2^ + 0.007*z*^3^	0.990	0.360
Hutoumen	*C* = 0.309*z* − 0.036*z*^2^ + 0.002*z*^3^	0.993	0.302

**Table 4:** The release rates and fluxes of NH_4_^+^-N and DRP in sediments of Daya Bay.

Factor	Site	D_s_ × 10^−6^/(cm^2^∙s^−1^)	*φ*_0_	F/(mg∙m^−2^∙d^−1^)	Area/m^2^	*M/*(t∙a^−1^)
NH_4_^+^-N	Aotou	10.30	0.75	371.070	100	13.544
	Shenshuigang	9.15	0.618	142.759	100	5.211
	Hutoumen	8.85	0.488	15.254	100	0.557
DRP	Aotou	4.10	0.75	9.211	100	0.336
	Shenshuigang	3.64	0.618	0.700	100	0.026
	Hutoumen	3.52	0.488	0.448	100	0.016

It can be found from Table 4 that the release rates of nitrogen and phosphorus in Daya Bay are not ignorable. As previous analysis data shows, the release rate in Aotou is the highest. Ammonia nitrogen and DRP release rates reach up to 371 mg/(m^2^∙d) and 9.211 mg/(m^2^∙d), respectively, which is much higher than the research results for Fubao Bay sediments in Dianchi Lake (where ammonia nitrogen is about 160 mg/(m^2^·d) and DRP is about 4.0 mg/(m^2^·d)) measured by Li Bao *et al*. [15], indicating that the nitrogen and phosphorus pollution in the aquaculture area of Daya Bay is pretty serious. On the one hand, sediments in the aquaculture area provide nutrients to the water body and maintain the primary productivity in Daya Bay [16]. On the other hand, the release rates of nitrogen and phosphorus shown in Table 4 are so large that they will have an effect on water body for several years, even if the nutrients from outer sources are controlled effectively. Aquaculture-cages exist widely distributed on the sea surface of Daya Bay. With the growing demand for seafood, the aquaculture plants may be expected to increase both in number and scale. Given this, in order to meet human demands for seafood, appropriate environmental management and planning actions must be taken to avoid an irreversible impact on the aquatic environment.

### 3.5. Diffusion and Transport of Nutrients in Seawater at the Area of Aquaculture

From the above, we know that the concentration of nutrients released by the sediments from 100 m^2^ to aquaculture-cages is far larger than the background concentration in seawater. Thanks to the movement of rising tide, falling tide and ocean currents, the pollutants released to seawater by the sediments are diffused, diluted and mixed so that the concentration of pollutants is reduced [17]. Generally, aquaculture-cages are floating on the sea year after year, creating nutrient-rich sediments and releasing them to seawater. Based on the theory of environmental hydraulics [18], sediments from each aquaculture-cage can be regarded as a point source of horizontal two-dimensional turbulent diffusion with equal-strength constant in unbounded space. Furthermore, nutrients are assumed to be conservative after being released on sediment-water interface. Given that Daya Bay has the property of tide and the flow velocity is to-and-fro with time, which make the flow field and concentration field complex, so mathematical models are the most suitable tool for the studies of the diffusion and transport on pollution [19]. 

**Figure 7:**
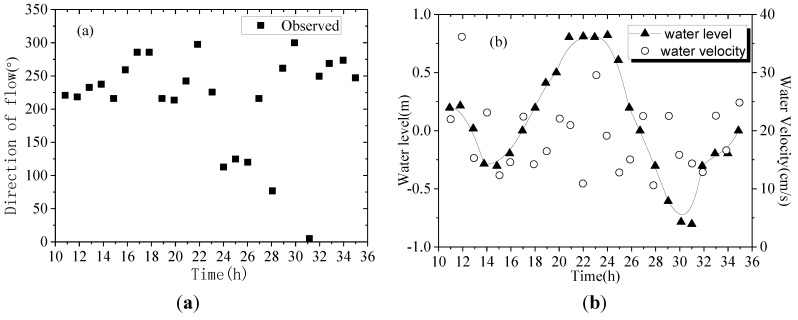
Observed flow velocities, flow directions and water levels closed to Hutoumen in Daya Bay.

A two-dimensional mathematical model is feasible for numerical simulation in Daya Bay, since the surface width here is much larger than water depth. Equations in two-dimensional depth-averaged unsteady shallow water are shown in the literature [20] and the transport equation of pollutants is given as Equation (3):

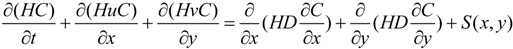
(3)
where *C*(*x*, *y*) is the horizontal two-dimensional concentration of nutrients [ML^−3^], *u* is the velocity of water in longitudinal direction [LT^−1^], *v* is the velocity of water in transverse direction [LT^−1^], *t* is time [T], *D* is mixing coefficient [L^2^T^−1^], *H* is water depth, *x* is the displacement from sediments-released point along tidal direction, *y* is the lateral displacement from sediments-released point, *S* is source intensity which means the pollutant mass discharged per unit time and per unit area [MT^−1^L^−2^] and can be found in the fifth column of Table 4. The mixing coefficient can be obtained using Equation (4) [18]:

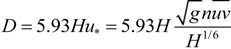
(4)
Where *n* is the Manning roughness of seabed and *uv* is the resultant velocity of *u* and *v*. According to Equations (3) and (4), the nutrient concentration and extent of pollution caused by the sediments released from aquaculture-cages can be calculated. Mixing and diffusion of pollutants in water are related to flow velocity and wind velocity. From 11 a.m. on 21 November 2002 to 11 a.m. on 22 November 2002, the resultant velocity and water level under the movement of tidal undulation and wind near Hutoumen were determined by Wang *et al*. [20] and are shown in Figure 7.

**Figure 8:**
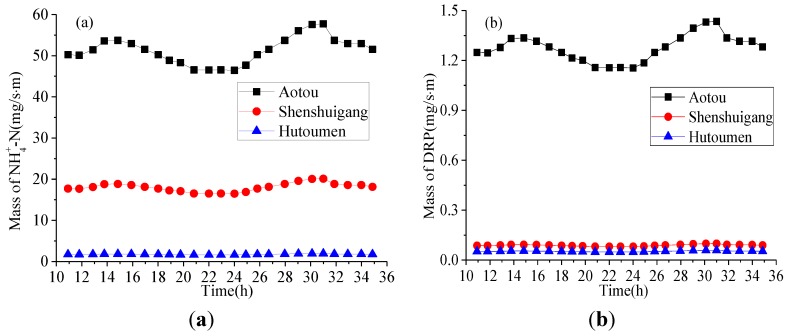
Changes of nutrient magnitude with time in sediments sampled in the three sites.

**Figure 9:**
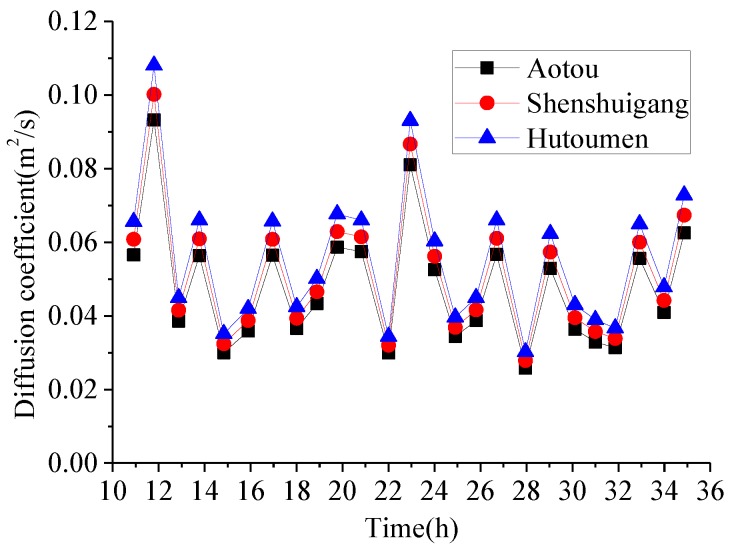
Changes of diffusion coefficients with time in sediments sampled in the three sites.

According to the theory of tidal dynamics in Daya Bay, the characteristic tidal hydrodynamics possess good periodicity. As Aotou and Shenshuigang are not far from Hutoumen (about 5 km), the tidal hydrodynamic forces at Aotou and Shenshuigang were assumed to have similar characteristics to those in Hutoumen. The Manning roughness of the seabed in the three sites is set as 0.025 [21]. Combining the diffusion fluxes in Table 4, water depth in Table 1 and Equation (4), the diffusion coefficient and the mass of nutrients discharged within one day and night can be calculated as shown in Figure 8 and Figure 9. 

From Figure 8, we can see that the mass of nutrients discharged per unit time and per unit water depth varies with tides, but the amplitude of the variation is rather small. The quantities of nutrients released in the three sites are different and the largest one is at Aotou. As shown in Figure 9, the diffusion coefficients at the three sites show a large amplitude of variation within one day, but they are similar at the same time, indicating that the diffusion coefficient is mainly related to the velocity and water depth. That is to say, the diffusion coefficient of different nutrients remains uniform at the same seawater area. 

**Figure 10:**
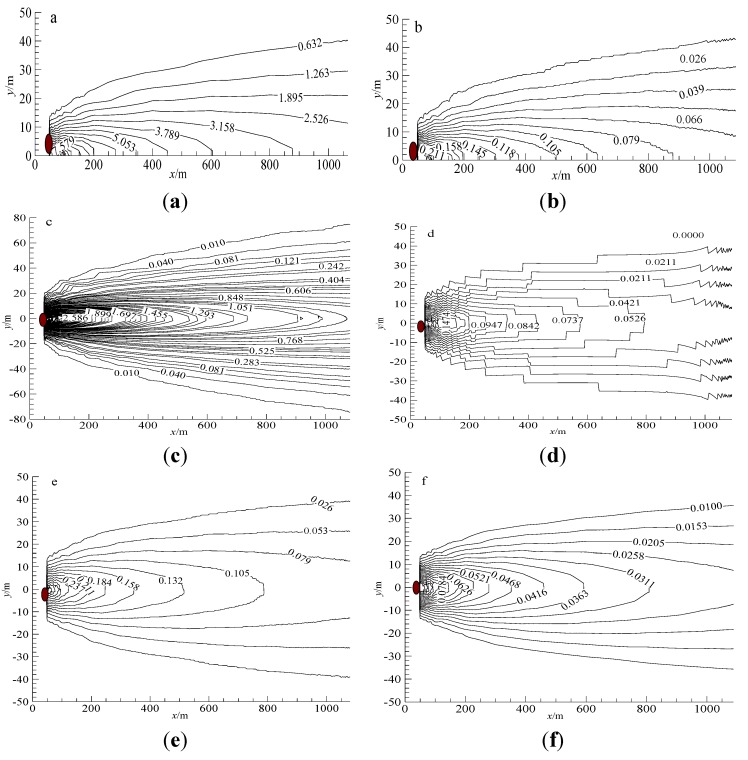
Diffusion distributions of nutrients in seawater (Unit: mg/L) (**a**) NH_4_^+^-N at Aotou site; (**b**) DRP at Aotou site; (**c**) NH_4_^+^-N at Shenshuigang site; (**d**) DRP at Shenshuigang site; (**e**) NH_4_^+^-N at Hutoumen site; (**f**) DRP at Hutoumen site.

Under the impulse of tide in Daya Bay within one day, the nutrients released from the sediment-water interface ate the three sites are diffused and mixed as shown in Figure 10. Among them, the red areas refer to point sources from which sediments are released, and the contour lines represent nutrient concentrations on the water surface. The diffusion and transport of nutrients at Aotou, which lies on the off-shore of Daya Bay, are influenced by reflection from the shore-side. However, the contrary is the case in Shenshuigang and Hutoumen. The diffusion and transport of pollutants are distributed symmetrically in the transverse direction, since Shenshuigang and Hutoumen are located in the area far away from seacoast in Daya Bay. As shown in Figure 10(a,b), due to the combined effect of pollution from residents and aquaculture-cages, the concentrations of nitrogen and phosphorus released from sediments remain high on the water surface at Aotou. The concentration of NH_4_^+^-N is not less than 2.53 mg/L within 1 km away from this site along the tidal direction and stays at 0.62 mg/L within 50 m away along lateral direction, with a strip zonal distribution, far greater than the values set in the sea water quality standard (GB3097-1997).

As found in field investigation, owing to the numerous aquaculture cages and a small town nearby, the pollution at Aotou is pretty serious and the sea water there is black and smelly. The ratio of NH_4_^+^-N concentration and DRP concentration is about 40:1. According to a new classification standard of eutrophication and corresponding evaluation model based on N/P value put forward by Guo *et al*. [22], caused by nitrogen and phosphorus released from sediments only, the eutrophication class level in Aotou is V_p_-level (namely medium restrictive potential eutrophication level of phosphorus). This indicates that as long as a suitable amount of phosphorus is supplemented in water body, this part of phosphorus will come into play and result in eutrophication. 

As shown in Figure 10(c,d), the diffusion of nitrogen and phosphorus on the water surface of Shenshuigang are distributed like a strip. The concentrations of NH_4_^+^-N and DRP are greater than 1 mg/L and 0.5 mg/L, respectively, within 1 km away from this station along the tidal direction. They are greater than 0.2 mg/L and 0.02 mg/L respectively within 50 m away along lateral direction. They are all greater than the second standard of the sea water quality (GB3097-1997). The ratio of NH_4_^+^-N concentration and DRP concentration is about 20:1, being classed as III-level (namely eutrophication).

The nutrient concentration at Hutoumen is the lowest. As shown in Figure 10(e,f), the concentrations of NH_4_^+^-N and DRP are greater than 0.08 mg/L and 0.025 mg/L, respectively, within 1 km away from this station along the tidal direction. They are greater than 0.02 mg/L and 0.01 mg/L, respectively, within 50 m away along lateral direction. Based on the sea water quality standards (GB3097-1997) (where the concentration of non-ionic nitrogen should not be greater than 0.02 mg/L and that of labile phosphorus no greater than 0.03 mg/L), we found that the pollution caused by the sediments in Hutoumen are distributed as a strip with a width of 38 m and a length of 1,100 m. The ratio of NH_4_^+^-N concentration and DRP concentration is about 3:1, being classed as *VI*_N_-level (namely restrictive potential eutrophication level of nitrogen). This indicates that as long as a suitable amount of nitrogen is supplemented in water body, this part of nitrogen will come into play and result in eutrophication.

The analysis above is under an assumption that the outer pollution source has been cut off. The impact on environment is caused by nutrients released from the sediments in the seabed only. It can be found that the effect on water environment caused by the sediments in the aquaculture area of Daya Bay is rather serious. The pollution area caused by each aquaculture-cages is no less than 0.04 km^2^ (0.038 × 1.1). Different aquaculture-cages show different restrictive potential eutrophication of nitrogen and phosphorus, indicating that whichever is released exorbitantly, nitrogen or phosphorus, is likely to bring about eutrophication to water body. With the rapid development of aquaculture cage fisheries in Daya Bay, the impacts of pollution will inevitably lead to some more severe water environmental problems.

## 4. Conclusions

In this paper, the physical properties of the sediments and nutrients content at three aquaculture sites of Daya Bay were determined, and the fluxes of nutrients across sediment-water interface and the polluted area were calculated. We can draw the following conclusions:

(1) Due to the high concentrations of nitrogen and phosphorus, the aquaculture area in Daya Bay has become a potential source of inner pollution.

(2) Based on the classical Fick law of diffusion, the release rates and annual release quantities of ammonia nitrogen and active phosphate on the sediment-water interface of three sites were calculated. For ammonia nitrogen, the annual release quantities across water and sediment interface at Aotou, Shenshuigang and Hutoumen are 13.5 t/a, 5.2 t/a and 0.56 t/a, respectively. For active phosphate, they are 0.34 t/a, 0.03 t/a and 0.02 t/a, respectively. These indicate that the sediments from aquaculture contribute a lot to the nitrogen and phosphorus contents in seawater.

(3) Considering the effect of tide, wind and water depth, the diffusion and transport of ammonia nitrogen and active phosphate after being released on the sediment-water interface were computed using the theory of environmental hydraulics. It can be found that the concentration distribution of ammonia nitrogen and phosphate appears as a strip with a width of 50 m and a length of 1 km. Different degrees of eutrophication exist in the sea areas near the three sites. With the rapid development of aquaculture cages in Daya Bay, the impacts of pollution dominate and will inevitably lead to some more severe water environmental problems.
